# Bronchopleural communication following intrapleural doses of tPA/DNase for empyema

**DOI:** 10.1002/rcr2.646

**Published:** 2020-08-31

**Authors:** Bapti Roy, Mark C. Teh, Yi Jin Kuok, Y. C. Gary Lee

**Affiliations:** ^1^ Department of Respiratory Medicine Sir Charles Gairdner Hospital Perth WA Australia; ^2^ Department of Radiology Sir Charles Gairdner Hospital Perth WA Australia; ^3^ Centre for Respiratory Health School of Medicine, University of Western Australia Perth WA Australia; ^4^ Department of Medicine University of Hong Kong Hong Kong

**Keywords:** Bronchopleural, deoxyribonuclease, empyema, pleural infection, tissue plasminogen activator

## Abstract

Intrapleural tissue plasminogen activator (tPA) and deoxyribonuclease (DNase) therapy is a new treatment for pleural infection. Clinical experiences of tPA/DNase therapy, and its complications, are cumulating. We present a patient with multiloculated empyema but no initial evidence of a bronchopleural fistula. She was treated with antibiotics and chest tube drainage of the basal collection through which four doses of tPA/DNase were delivered with success. The lateral collection worsened necessitating separate tube drainage and tPA/DNase treatment. She reported chest “fullness” when instilled the second dose. The third instillation of tPA triggered immediate vigorous coughing and expectoration of salty‐tasting fluid, likely the tPA/saline solution. The symptoms spontaneously settled after 15 min, with no evidence of air leak. The loculated fluid was successfully evacuated. The patient made a full recovery after an antibiotic course with no long‐term consequences. Pulmonary migration of drugs via a bronchopleural communication, although rare, can occur with intrapleural tPA/DNase therapy.

## Graphical abstract

Pulmonary migration of intrapleural drugs via a bronchopleural communication is a rare and poorly described complication of intrapleural tissue plasminogen activator/deoxyribonuclease (tPA/DNase) therapy. We report the first detailed case of a bronchopleural communication after intrapleural tPA/DNase instillation. Despite this, the patient made an uneventful recovery from the pleural infection without requiring surgical drainage.

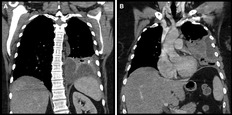

## Introduction

Pneumonia is common and necrosis of peripheral lung tissue can breach the visceral pleura and create a communication (or fistula) between the lung and pleural cavity.

Pleural infection is a common complication of pneumonia and usually requires chest tube drainage. Administration of intrapleural tissue plasminogen activator (tPA) and deoxyribonuclease (DNase) is increasingly used to facilitate pleural fluid evacuation [1, 2].

Pulmonary migration of intrapleural drugs via a bronchopleural communication is a rare and poorly described complication of intrapleural tPA/DNase therapy. We report the first detailed case of a bronchopleural communication after intrapleural tPA/DNase instillation. Despite this, the patient made an uneventful recovery from the pleural infection without requiring surgical drainage.

## Case Report

**Figure 1:**
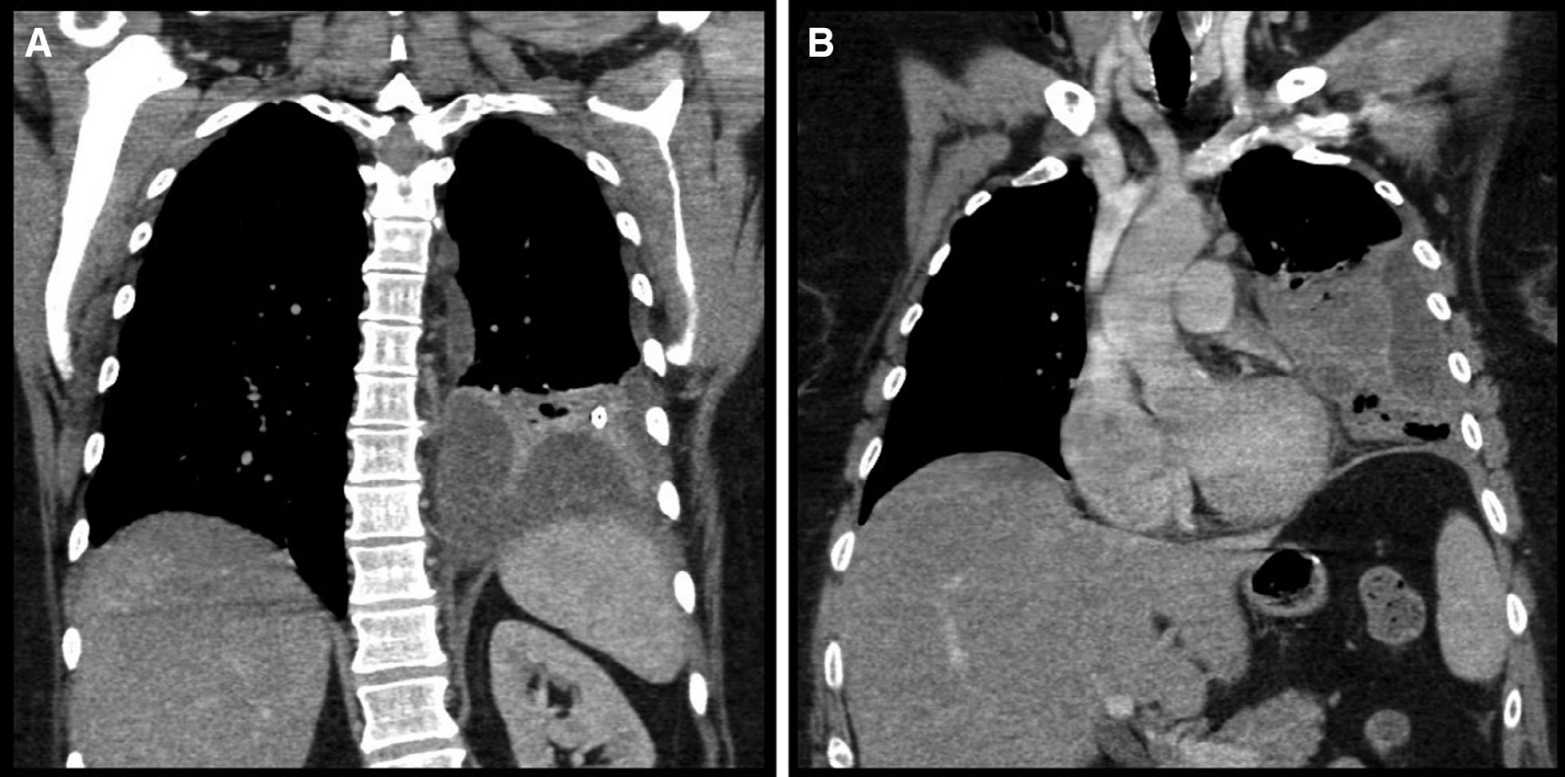
Computed tomography (CT) images at initial presentation demonstrating separate (A) basal and (B) lateral pleural fluid collections.

A 60‐year‐old female with a past history of gastric sleeve surgery and hiatus hernia presented to an outside hospital with fever and left‐sided chest discomfort, leucocytosis (25 × 10^9^/L), and raised C‐reactive protein (CRP) (443 mg/L). Computed tomography (Fig. 1) and ultrasonography demonstrated left‐sided pneumonia, a large basal multiloculated effusion, and separate lateral and anterior pleural fluid collections. She was treated with intravenous piperacillin‐tazobactam and a Seldinger chest tube which failed to drain any fluid. She had ongoing high fever and inflammatory markers, and was transferred to our pleural service for further management.

The initial drain was removed. A new 12‐F drain was inserted under imaging guidance into the basal locule and removed 100 mL of pleural fluid (pH: 7.10 and lactate dehydrogenase (LDH): 4660 IU/L) but no organisms were cultured. Antibiotics were escalated to meropenem. No further fluid was drained overnight, and intrapleural tPA (2.5 mg) and DNase (5 mg) therapy was started. Her fever settled quickly. After four doses of tPA/DNase, a total of 1325 mL of fluid was drained, with near‐complete resolution of the basal locule. CRP improved (170 mg/L) and her drain was removed.

**Figure 2:**
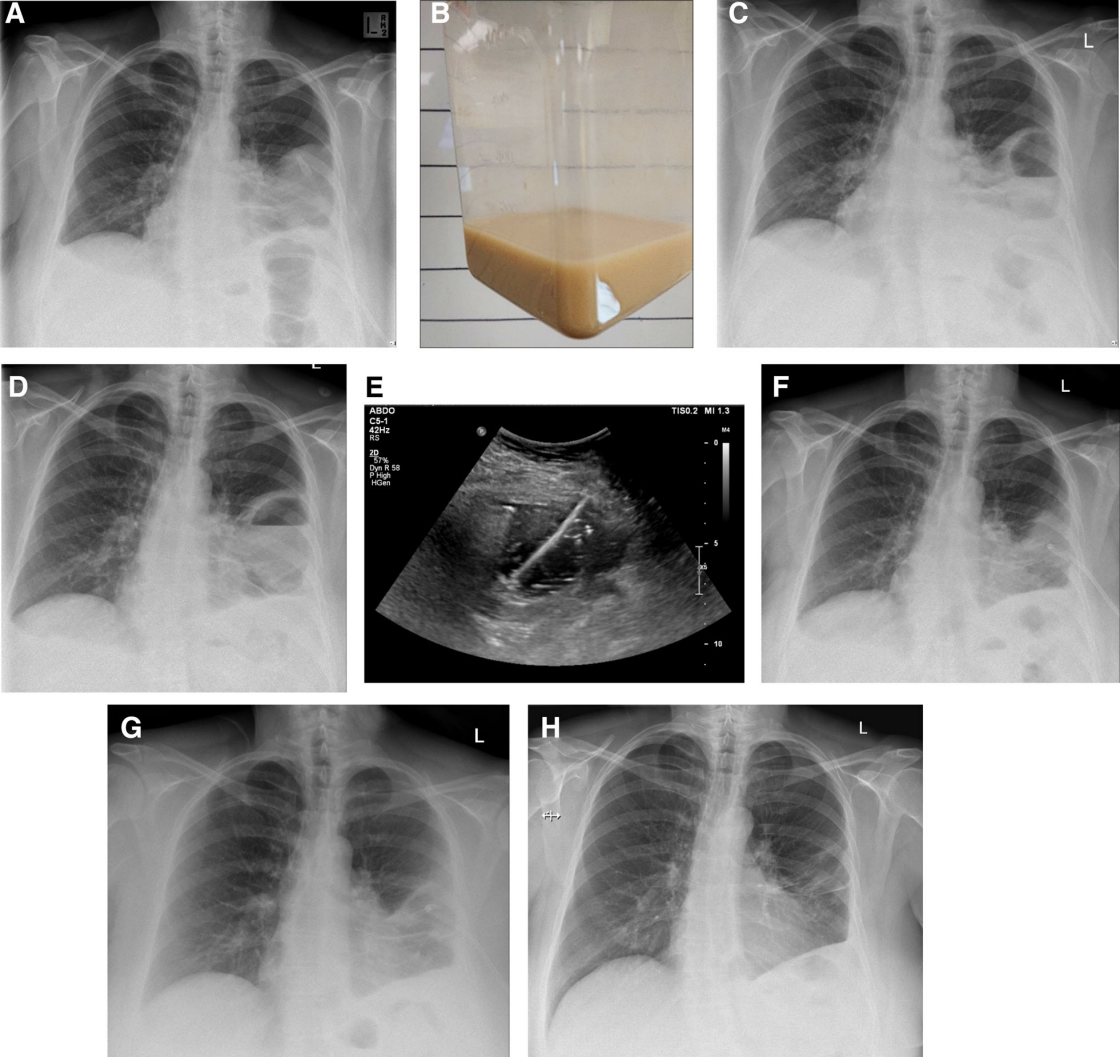
(A) The basal collection cleared with tissue plasminogen activator/deoxyribonuclease (tPA/DNase) therapy but the lateral locule enlarged with time and (B) frank pus was aspirated via a 5‐F catheter. However, the fluid reaccumulated over the following few days (C). A 12‐F chest drain was inserted under real‐time imaging guidance and Seldinger technique (D). The position of the drain was confirmed with ultrasound (US) (E) and chest X‐ray (CXR) (F). The chest radiograph showed near‐complete clearance of the lateral locule after the incidence of migration of the intrapleural tPA dose to the lung and subsequent significant coughing (G). Follow‐up radiograph after three weeks demonstrated continued resolution of the collections (H).

During that time, the lateral locule had increased in size (Fig. 2A) and was aspirated under imaging guidance to near dryness. The frank pus (Fig. 2B) removed grew *Streptococcus constellatus* and mixed anaerobic bacteria. Fluid reaccumulated in the locule after five days and her fever recurred. A 12‐F intercostal catheter (Fig. 2C–F) was inserted under real‐time ultrasound guidance and drained 120 mL of turbid fluid initially with no evidence of air leak via the tube. Because of significant residual fluid, a course of intrapleural tPA (2.5 mg) and DNase (5 mg) was initiated to this lateral collection. The first dose of tPA/DNase was administered uneventfully and drained 75 mL. The patient complained of a sensation of chest “fullness” during instillation of the second dose. Upon the third instillation of tPA, she described a bubbling sensation in her chest, followed immediately by violent coughing and expectoration of salty‐tasting fluid, likely the tPA solution which was diluted in saline. Her coughing resolved spontaneously within 15 min. No bubbling in the chest drain occurred at any time. Subsequent radiograph showed significant clearance of the loculated fluid (Fig. 2G). No further doses of fibrinolytic therapy were given and the chest tube was removed. In total, 550 mL was drained from the lateral locule. She had no further fever and CRP decreased to 90 mg/L.

The patient was discharged with intravenous, followed by oral antibiotics. She made a full recovery, and returned to full‐time work. Follow‐up imaging showed continual resolution of her lung consolidation and the small residual effusion (Fig. 2H). There has been no clinical or radiological evidence of air leak during the two months of follow‐up.

## Discussion

We provided the first detailed description of uncovering of a bronchopleural communication in the course of intrapleural tPA/DNase therapy in a patient with no clinical or radiological evidence of a bronchopleural fistula.

Combined intrapleural tPA/DNase therapy has been shown to cure most patients of pleural infection without requiring surgery [1, 2]. Administration is generally considered safe; chest pain and non‐fatal pleural bleeding are recognized complications [1, 2]. The optimal dose of tPA is still under investigation [3].

Bronchopleural communications are common with severe pneumonia and may be small and not detectable clinically or on CT. Several mechanisms may explain how intrapleurally administered tPA could migrate into the lung. First, the fibrinolytic properties of tPA/DNase may have opened a previously healed fistula. Second, tPA/DNase is potent at breaking down adhesions [4]. In so doing, it may allow any existing fistula that is confined with a loculated fluid collection to communicate with the pleural cavity. Third, tPA/DNase may have contributed to the development of a new bronchopleural communication by lysing fragile/necrotic peripheral lung tissues. Fourth, we cannot exclude an iatrogenic puncture of the lung during prior pleural interventions.

Our patient developed symptoms only at the second and third doses. Multiple doses of intrapleural tPA, together with pressure generated from fluid instillation to a small cavity, may have triggered any of the above‐mentioned mechanisms, facilitating drug migration into the lung.

Clinicians are often concerned with the possibility of creating bronchopleural communication with fibrinolytic therapy in empyema patients with peripheral lung abscesses. No previous study has specifically described the development of a bronchopleural communication during intrapleural tPA/DNase therapy or its clinical consequences [2, 5]. Our case highlights that a bronchopleural communication may develop anytime during the tPA/DNase course, and without prior evidence of air leak even when connected to underwater seal drainage. A bubbling sensation within the chest and coughing during instillation (with or without drug expectoration) should alert clinicians to this complication. Although unpleasant, the discomfort is short lived and unlikely to generate long‐term damage. Our patient had no noticeable long‐term sequelae on follow‐up reviews.

### Disclosure Statement

Appropriate written informed consent was obtained for publication of this case report and accompanying images.
